# Supplementary material for: Mitochondrial Genomes of Hestina persimilis and Hestinalis nama (Lepidoptera, Nymphalidae): Genome Description and Phylogenetic Implications
Source: Insects. 2021 Aug 20;12(8):754. doi: 10.3390/insects12080754 (PMC8397171; doi:10.3390/insects12080754)
Supplement: Supplementary file 1 [file insects-12-00754-s001.zip › insects-1320628-supplementary.pdf]

Table S1. Sequences of 22 pairs of primers.

No. of fragment	Primer name	Nucleotide sequence (5′-3′)
1	TMJ-210	5′ -ATAAAAGCTTTTGGGCTCATACCT
	TY-N1433	5′ -GGCTGAATAATAAGCGATAAATTGTAAA
2	TW-J1301	5′ -GTTAAAWTAAACTAATARTCTTCAAA
	C1-N2353	5′ -GCTCGTGTATCAATATCTATWCC
3	C1-J2195	5′ -TGATTTTTTGGWCATCCWGAAGT
	C1-N2776	5′ -GATAATCTGAATATCGWCGNGG
4	C1-J2756	5′ -ACATTTTTTCCACARCAYTT
	C2-N3389	5′ -TATTCATARCTTCARTATCATTG
5	TL2-J3043	5′ -GGCAGACTATATGYAATGRATTTAA
	TK-N3796	5′ -ACTATAAAATGGTTTAAGAG
6	TK-J3790	5′ -CATTAGATGACTGAAAGCAAGTA
	A6-N4552	5′ -ATGTCCWGCAATTATGTTWGC
7	A6-J4463	5′ -TTTATTCATATWATWCCNCAAGG
	C3-N4908	5′ -CGTGAAAYATCTCGTCATCATTG
8	C3-J4792	5′ -GTAGATTATAGACCWTGRCC
	N3-N5731	5′ -TTTGGATCAAACCCRCAYTC
9	C3-J5470	5′ -GCTGCAGCYTGATAYTGRCA
	TF-N6384	5′ -ATATTATAGAGYTTRAYAYTGAAG
10	TN-J6172	5′ -AGAGGTATATCACTGTTAATGA
	N5-N7211	5′ -TTAAAGCTTTAYTATTTATRTGYGC
11	N5-J7077	5′ -TTAAATCTTTWGARTAAAAYCC
	N5-N7793	5′ -TTGGGTTGRGATGGNYTAGG
12	N5-J7572	5′ -AAAAGGAATTTGAGCACTTTTWGT
	N4-N8727	5′ -AAATCTTTRATTGCTTATTCWTC
13	N4-J8641	5′ -CCAGAAGAACATAANCCRTG
	N4L-N9629	5′ -GTTTGTGAAGGWTYTTTRGG
14	N4-J9172	5′ -CGCTCTGGYTGRTAACCYCA
	CB-N11010	5′ -TATCTACAGCRAATCCYCCYCA
15	CB-J10621	5′ -TTCTTCTYRATGAAAYATAGGWTC
	N1-N12067	5′ -AATCGWACTCCWTTTGATTTTGC
16	N1-J11876	5′ -CGAGGTAAAGTMCCWCGAACYCA

	N1-N12595	5′-GTWGCTTTTTTGACTTTATTRGARCG
17	N1-J12261	5′-TACTTCATAAGAAATAGTYTGRGC
	LR-N13000	5′-TTACCTTAGGGATAACAGCGTAA
18	LR-J12888	5′-CCGGTTTGAACTCARATCATGTA
	LR-N13889	5′-ATTTATTGTATCTTKTGTATCAG
19	LR-J13342	5′-CCTTTGTACRGTCAAAATACYGC
	SR-N14220	5′-ATATGYACAYATTGCCCGTC
20	SR-J14197	5′-TACCYCTACTTTGTTACGACTT
	SR-N14745	5′-GTGCCAGCAGYYGCGGTTANAC
21	SR-J14610	5′-ATAATAGGGTATCTAATCCTAGT
	TM-N200	5′-TCCTTTATATRTGAGGTATGARCC
22	TI-J34	5′-GCCTGAATAAAGGRTTAYYCTGATA
	N2-N993	5′-GGTATAAAACCCATAAATGGNGG

Table S2. List of species used to construct the phylogenetic tree.

Family	Subfamily	species	GenBank accession no.	Length (bp)
Nymphalidae	Apaturinae	*Hestina persimilis*	MT110153	15,252
Nymphalidae	Apaturinae	*Hestina nama*	MT110154	15,208
Nymphalidae	Apaturinae	*Hestina assimilis*	MN182752	15,262
Nymphalidae	Apaturinae	*Apatura laverna*	MF444860	15,187
Nymphalidae	Apaturinae	*Herona marathus*	KT279805	15,487
Nymphalidae	Apaturinae	*Euripus nyctelius*	KR020515	15,417
Nymphalidae	Apaturinae	*Sasakia funebris*	JX131328	15,233
Nymphalidae	Apaturinae	*Apatura metis*	JF801742	15,236
Nymphalidae	Apaturinae	*Chitoria ulupi*	KP284554	15,279
Nymphalidae	Apaturinae	*Apatura ilia*	JF437925	15,242
Nymphalidae	Apaturinae	*Timelaea maculata*	KC572131	15,178
Nymphalidae	Apaturinae	*Sasakia charonda*	JX119051	15,233
Nymphalidae	Biblidinae	*Hamadryas epinome*	KM378244	15,207
Nymphalidae	Calinaginae	*Calinaga davidis*	HQ658143	15,267
Nymphalidae	Danainae	*Parantica sita*	MG571524	15,156
Nymphalidae	Danainae	*Tirumala limniace*	KJ784473	15,285
Nymphalidae	Danainae	*Ideopsis similis*	KJ476729	15,200
Nymphalidae	Danainae	*Danaus plexippus*	KC836923	15,314
Nymphalidae	Danainae	*Euploea midamus*	KJ866207	15,187
Nymphalidae	Heliconiinae	*Damora sagana*	KY971464	15,151
Nymphalidae	Heliconiinae	*Acraea issoria*	GQ376195	15,245
Nymphalidae	Heliconiinae	*Argynnis hyperbius*	JF439070	15,156
Nymphalidae	Heliconiinae	*Issoria lathonia*	HM243590	15,172
Nymphalidae	Heliconiinae	*Fabriciana nerippe*	JF504707	15,140
Nymphalidae	Heliconiinae	*Cethosia biblis*	KR066948	15,286
Nymphalidae	Heliconiinae	*Heliconius cydno*	KM208636	15,367
Nymphalidae	Libytheinae	*Libythea celtis*	HQ378508	15,164
Nymphalidae	Limenitidinae	*Neptis alwina*	MG457128	15,226
Nymphalidae	Limenitidinae	*Athyma sulpitia*	JQ347260	15,268
Nymphalidae	Limenitidinae	*Limenitis camilla*	MG747618	15,129
Nymphalidae	Limenitidinae	*Tacola eulimene*	MG747616	15,669
Nymphalidae	Nymphalinae	*Salamis anteva*	MH917707	15,201
Nymphalidae	Nymphalinae	*Precis andremiaja*	MH917706	15,239
Nymphalidae	Nymphalinae	*Vanessa indica*	MG736927	15,191
Nymphalidae	Nymphalinae	*Polygonia c-aureum*	MF407452	15,209
Nymphalidae	Nymphalinae	*Kallima inachus*	HM243591	15,150
Nymphalidae	Nymphalinae	*Junonia iphita*	KU577290	15,190
Nymphalidae	Nymphalinae	*Melitaea cinxia*	GQ398377	15,170
Nymphalidae	Satyrinae	*Ninguta schrenckii*	KF881052	15,261
Nymphalidae	Satyrinae	*Lethe dura*	KF906485	15,259
Nymphalidae	Satyrinae	*Elymnias hypermnestra*	KF906484	15,167
Nymphalidae	Satyrinae	*Callerebia suroia*	KF906483	15,208
Nymphalidae	Satyrinae	*Triphysa phryne*	KF906487	15,143
Nymphalidae	Satyrinae	*Melanargia asiatica*	KF906486	15,142
Nymphalidae	Satyrinae	*Melanitis leda*	JF905446	15,122

Nymphalidae	Satyrinae	*Mycalesis intermedia*	MN610565	15,386
Nymphalidae	Satyrinae	*Lasiommata deidamia*	MG880214	15,244
Nymphalidae	Satyrinae	*Hipparchia autonoe*	GQ868707	15,489
Nymphalidae	Satyrinae	*Davidina armandi*	KF881046	15,214
Nymphalidae	Satyrinae	*Stichophthalma louisa*	KP247523	15,721
Nymphalidae	Satyrinae	*Pararge aegeria*	KJ547676	15,240
Nymphalidae	Satyrinae	*Neope muirheadii*	MN242789	15,217
Nymphalidae	Satyrinae	*Ypthima motschulskyi*	MN242788	15,232
Nymphalidae	Satyrinae	*Minois dryas*	MN242787	15,194
Papilionidae	Papilioninae	*Papilio protenor*	KY272622	15,268
Papilionidae	Papilioninae	*Lamproptera curius*	KJ141168	15,277

Table S3. The starting partitions used to initiate the PartitionFinder analysis.

Dataset	starting partitions
13PCG	Gene1_pos1 = 1-684\3;
	Gene1_pos2 = 2-684\3;
	Gene1_pos3 = 3-684\3;
	Gene2_pos1 = 685-858\3;
	Gene2_pos2 = 686-858\3;
	Gene2_pos3 = 687-858\3;
	Gene3_pos1 = 859-2409\3;
	Gene3_pos2 = 860-2409\3;
	Gene3_pos3 = 861-2409\3;
	Gene4_pos1 = 2410-3087\3;
	Gene4_pos2 = 2411-3087\3;
	Gene4_pos3 = 2412-3087\3;
	Gene5_pos1 = 3088-3876\3;
	Gene5_pos2 = 3089-3876\3;
	Gene5_pos3 = 3090-3876\3;
	Gene6_pos1 = 3877-5028\3;
	Gene6_pos2 = 3878-5028\3;
	Gene6_pos3 = 3879-5028\3;
	Gene7_pos1 = 5029-5973\3;
	Gene7_pos2 = 5030-5973\3;
	Gene7_pos3 = 5031-5973\3;
	Gene8_pos1 = 5974-6167\3;
	Gene8_pos2 = 5975-6167\3;
	Gene8_pos3 = 5976-6167\3;
	Gene9_pos1 = 6168-6524\3;
	Gene9_pos2 = 6169-6524\3;
	Gene9_pos3 = 6170-6524\3;
	Gene10_pos1 = 6525-7883\3;
	Gene10_pos2 = 6526-7883\3;
	Gene10_pos3 = 6527-7883\3;
	Gene11_pos1 = 7884-8174\3;
	Gene11_pos2 = 7885-8174\3;
	Gene11_pos3 = 7886-8174\3;
	Gene12_pos1 = 8175-9932\3;
	Gene12_pos2 = 8176-9932\3;
	Gene12_pos3 = 8177-9932\3;
	Gene13_pos1 = 9933-10472\3;
	Gene13_pos2 = 9934-10472\3;
	Gene13_pos3 = 9935-10472\3;

Gene1-13 represent *atp6, atp8, cox1, cox2, cox3, cob, nad1, nad2, nad3, nad4, nad4l, nad5, nad*6, respectively.

Table S4. Evolutionary models from Partition strategies start scheme used in the phylogenetic analysis.

**Subset**	**Best Model**	**# sites**	**Partition names**
1	GTR+I+G	228	Gene1_pos1
2	GTR+I+G	228	Gene1_pos2
3	GTR+I+G	228	Gene1_pos3
4	GTR+I+G	58	Gene2_pos1
5	GTR+G	58	Gene2_pos2
6	GTR+G	58	Gene2_pos3
7	GTR+I+G	517	Gene3_pos1
8	GTR+I+G	517	Gene3_pos2
9	GTR+G	517	Gene3_pos3
10	GTR+I+G	226	Gene4_pos1
11	GTR+I+G	226	Gene4_pos2
12	GTR+G	226	Gene4_pos3
13	GTR+I+G	263	Gene5_pos1
14	GTR+I+G	263	Gene5_pos2
15	GTR+G	263	Gene5_pos3
16	GTR+I+G	384	Gene6_pos1
17	GTR+I+G	384	Gene6_pos2
18	GTR+I+G	384	Gene6_pos3
19	GTR+I+G	315	Gene7_pos1
20	GTR+I+G	315	Gene7_pos2
21	GTR+G	315	Gene7_pos3
22	GTR+G	65	Gene8_pos1
23	GTR+I+G	65	Gene8_pos2
24	GTR+G	64	Gene8_pos3
25	GTR+I+G	119	Gene9_pos1
26	GTR+G	119	Gene9_pos2
27	GTR+I+G	119	Gene9_pos3
28	GTR+I+G	453	Gene10_pos1
29	GTR+I+G	453	Gene10_pos2
30	GTR+G	453	Gene10_pos3
31	GTR+G	97	Gene11_pos1
32	GTR+G	97	Gene11_pos2
33	GTR+G	97	Gene11_pos3
34	GTR+I+G	586	Gene12_pos1
35	GTR+I+G	586	Gene12_pos2
36	GTR+G	586	Gene12_pos3
37	GTR+I+G	180	Gene13_pos1
38	GTR+I+G	180	Gene13_pos2
39	GTR+G	180	Gene13_pos3

Gene1-13 represent *atp6, atp8, cox1, cox2, cox3, cob, nad1, nad2, nad3, nad4, nad4l, nad5, nad*6, respectively.

Table S5. Bias of base composition of protein-coding genes in the two mitogenomes.

Hestina persimilis	**A%**	**T%**	**G%**	**C%**	**A+T%**	**G+C%**	**AT-skew**	**GC-skew**
All genes								
1st	35.6	46.3	9.5	8.5	81.9	18	-0.13065	0.05556
2nd	35.0	43.7	11.0	10.3	78.7	21.3	-0.11055	0.03286
3rd	30.7	47.6	10.7	11.0	78.3	21.7	-0.21584	-0.01382
Total	33.8	45.9	10.4	9.9	79.7	20.3	-0.15182	0.02463
Genes encoded on J-strand								
1st	40.1	43.5	7.7	8.6	83.6	16.3	-0.04067	-0.05521
2nd	30.5	40.5	13.5	15.5	71.0	29.0	-0.14085	-0.06897
3rd	31.3	49.7	6.7	12.3	81.0	19.0	-0.22716	-0.29474
Total	34.0	44.6	9.3	12.1	78.6	21.4	-0.13486	-0.13084
Genes encoded on N-strand								
1st	39.9	43.2	12.3	4.6	83.1	16.9	-0.03971	0.45562
2nd	24.4	49.0	15.8	10.8	73.4	26.6	-0.33515	0.18797
3rd	36.0	51.7	8.4	3.9	87.7	12.3	-0.17902	0.36585
Total	33.4	47.9	12.2	6.4	81.3	18.6	-0.17835	0.31183

Hestina nama	A%	T%	G%	C%	A+T%	G+C%	AT-skew	GC-skew
All genes								
1st	34.1	45.1	10.7	10.0	79.2	20.7	-0.13889	0.03382
2nd	33.3	43.4	12.3	11.0	76.7	23.3	-0.13168	0.05579
3rd	30.9	45.9	11.2	12.0	76.8	23.2	-0.19531	-0.03448
Total	32.8	44.8	11.4	11.0	77.6	22.4	-0.15464	0.01786
Genes encoded on J-strand								
1st	39.5	40.4	8.8	11.4	79.9	20.2	-0.01126	-0.12871
2nd	30.7	40.0	13.5	15.8	70.7	29.3	-0.13154	-0.07850
3rd	31.8	47.3	7.0	14.0	79.1	21.0	-0.19595	-0.33333
Total	34.0	42.6	9.7	13.7	76.6	23.4	-0.11227	-0.17094
Genes encoded on N-strand								

1st	36.1	44.3	14.8	4.7	80.4	19.5	-0.10199	0.51795
2nd	23.8	48.4	16.7	11.2	72.2	27.9	-0.34072	0.19713
3rd	32.8	52.4	10.7	4.1	85.2	14.8	-0.23005	0.44595
Total	30.9	48.4	14.1	6.7	79.3	20.8	-0.22068	0.35577

Table S6. Codon usage of protein genes of twelve mitogenomes in Apaturinae.

**Codon usage of protein genes in *Apatura ilia* mitogenome**											
**Domain: Data**											
Codon	Count	RSCU	Codon	Count	RSCU	Codon	Count	RSCU	Codon	Count	RSCU
UUU(F)	26.5	1.82	UCU(S)	8.2	1.96	UAU(Y)	12.6	1.76	UGU(C)	2.3	1.76
UUC(F)	2.7	0.18	UCC(S)	0.7	0.17	UAC(Y)	1.7	0.24	UGC(C)	0.3	0.24
UUA(L)	34.7	1.89	UCA(S)	7.7	1.85	UAA(*)	0	0	UGA(W)	6.8	1.89
UUG(L)	1.9	0.11	UCG(S)	0.1	0.02	UAG(*)	0	0	UGG(W)	0.4	0.11
CUU(L)	2.9	2.45	CCU(P)	4.8	2.1	CAU(H)	4.3	1.65	CGU(R)	1.2	1.21
CUC(L)	0.3	0.26	CCC(P)	1.4	0.61	CAC(H)	0.9	0.35	CGC(R)	0.2	0.15
CUA(L)	1.5	1.29	CCA(P)	2.8	1.22	CAA(Q)	4.1	1.83	CGA(R)	2.3	2.26
CUG(L)	0	0	CCG(P)	0.2	0.07	CAG(Q)	0.4	0.17	CGG(R)	0.4	0.38
AUU(I)	33.8	1.88	ACU(T)	5.9	2.07	AAU(N)	17.5	1.81	AGU(S)	2.7	1.24
AUC(I)	2.1	0.12	ACC(T)	0.8	0.3	AAC(N)	1.8	0.19	AGC(S)	0.2	0.07
AUA(M)	20.8	1.81	ACA(T)	4.7	1.64	AAA(K)	7.2	1.79	AGA(S)	5.8	2.65
AUG(M)	2.2	0.19	ACG(T)	0	0	AAG(K)	0.8	0.21	AGG(S)	0.1	0.04
GUU(V)	4.9	1.94	GCU(A)	5.2	2.37	GAU(D)	4.7	1.82	GGU(G)	4.1	1.08
GUC(V)	0.1	0.03	GCC(A)	0.6	0.28	GAC(D)	0.5	0.18	GGC(G)	0.9	0.24
GUA(V)	4.8	1.88	GCA(A)	2.8	1.31	GAA(E)	5.1	1.81	GGA(G)	7.8	2.07
GUG(V)	0.4	0.15	GCG(A)	0.1	0.04	GAG(E)	0.5	0.19	GGG(G)	2.3	0.61
Average# codons=285											
Codon usage of protein genes in *Apatura metis* mitogenome											
Domain: Data											
Codon	Count	RSCU	Codon	Count	RSCU	Codon	Count	RSCU	Codon	Count	RSCU
UUU(F)	27.5	1.84	UCU(S)	8	2.52	UAU(Y)	12.7	1.78	UGU(C)	2.3	1.82
UUC(F)	2.3	0.16	UCC(S)	0.8	0.24	UAC(Y)	1.5	0.22	UGC(C)	0.2	0.18
UUA(L)	33.7	4.97	UCA(S)	7.6	2.4	UAA(*)	0	0	UGA(W)	6.8	1.89
UUG(L)	2.2	0.33	UCG(S)	0.1	0.02	UAG(*)	0	0	UGG(W)	0.4	0.11
CUU(L)	2.8	0.41	CCU(P)	4.7	2.09	CAU(H)	4.2	1.62	CGU(R)	1.2	1.13
CUC(L)	0.4	0.06	CCC(P)	1.4	0.62	CAC(H)	1	0.38	CGC(R)	0.2	0.15
CUA(L)	1.6	0.24	CCA(P)	2.9	1.3	CAA(Q)	4.2	1.83	CGA(R)	2.5	2.42
CUG(L)	0	0	CCG(P)	0	0	CAG(Q)	0.4	0.17	CGG(R)	0.3	0.3
AUU(I)	33.5	1.88	ACU(T)	5.8	2.05	AAU(N)	17.6	1.82	AGU(S)	2.8	0.87
AUC(I)	2.2	0.12	ACC(T)	0.8	0.3	AAC(N)	1.7	0.18	AGC(S)	0.2	0.05
AUA(M)	20.8	1.82	ACA(T)	4.7	1.65	AAA(K)	7.2	1.77	AGA(S)	5.9	1.87
AUG(M)	2.1	0.18	ACG(T)	0	0	AAG(K)	0.9	0.23	AGG(S)	0.1	0.02
GUU(V)	5.2	2.02	GCU(A)	5.2	2.39	GAU(D)	4.7	1.82	GGU(G)	4.4	1.16
GUC(V)	0	0	GCC(A)	0.5	0.25	GAC(D)	0.5	0.18	GGC(G)	0.5	0.14
GUA(V)	4.8	1.86	GCA(A)	2.8	1.3	GAA(E)	5.2	1.81	GGA(G)	7.5	2
GUG(V)	0.3	0.12	GCG(A)	0.2	0.07	GAG(E)	0.5	0.19	GGG(G)	2.6	0.69
Average# codons=285											
Codon usage of protein genes in *Sasakia charonda* mitogenome											
Domain: Data											

Codon	Count	RSCU	Codon	Count	RSCU	Codon	Count	RSCU	Codon	Count	RSCU
UUU(F)	27.1	1.83	UCU(S)	7.8	2.44	UAU(Y)	13.2	1.83	UGU(C)	2.5	1.94
UUC(F)	2.5	0.17	UCC(S)	1.4	0.44	UAC(Y)	1.2	0.17	UGC(C)	0.1	0.06
UUA(L)	34.7	4.93	UCA(S)	7.1	2.22	UAA(*)	0	0	UGA(W)	6.8	1.87
UUG(L)	2.7	0.38	UCG(S)	0.3	0.1	UAG(*)	0	0	UGG(W)	0.5	0.13
CUU(L)	2.5	0.35	CCU(P)	4.8	2.12	CAU(H)	4.3	1.6	CGU(R)	1	1
CUC(L)	0.1	0.01	CCC(P)	1.5	0.68	CAC(H)	1.1	0.4	CGC(R)	0.2	0.15
CUA(L)	2.2	0.32	CCA(P)	2.7	1.2	CAA(Q)	4.3	1.87	CGA(R)	2.5	2.54
CUG(L)	0.1	0.01	CCG(P)	0	0	CAG(Q)	0.3	0.13	CGG(R)	0.3	0.31
AUU(I)	33.7	1.83	ACU(T)	5.3	1.9	AAU(N)	16.8	1.74	AGU(S)	1.9	0.6
AUC(I)	3.1	0.17	ACC(T)	0.8	0.3	AAC(N)	2.5	0.26	AGC(S)	0.4	0.12
AUA(M)	18.7	1.75	ACA(T)	4.9	1.77	AAA(K)	7.3	1.73	AGA(S)	6.1	1.91
AUG(M)	2.7	0.25	ACG(T)	0.1	0.03	AAG(K)	1.2	0.27	AGG(S)	0.5	0.17
GUU(V)	4.5	1.82	GCU(A)	5.4	2.33	GAU(D)	4.6	1.85	GGU(G)	3.8	1.02
GUC(V)	0.4	0.15	GCC(A)	0.9	0.4	GAC(D)	0.4	0.15	GGC(G)	0.7	0.19
GUA(V)	4.8	1.91	GCA(A)	2.5	1.1	GAA(E)	4.7	1.67	GGA(G)	7.8	2.09
GUG(V)	0.3	0.12	GCG(A)	0.4	0.17	GAG(E)	0.9	0.33	GGG(G)	2.6	0.7
Average# codons=286											
Codon usage of protein genes in *Sasakia funebris* mitogenome											
Domain: Data											
Codon	Count	RSCU	Codon	Count	RSCU	Codon	Count	RSCU	Codon	Count	RSCU
UUU(F)	27.6	1.87	UCU(S)	7.9	2.5	UAU(Y)	14.2	1.89	UGU(C)	2.5	1.88
UUC(F)	1.8	0.13	UCC(S)	1.1	0.34	UAC(Y)	0.8	0.11	UGC(C)	0.2	0.12
UUA(L)	36.7	5.18	UCA(S)	7.5	2.38	UAA(*)	0	0	UGA(W)	7	1.94
UUG(L)	1.5	0.21	UCG(S)	0.1	0.02	UAG(*)	0	0	UGG(W)	0.2	0.06
CUU(L)	3.1	0.43	CCU(P)	5.8	2.59	CAU(H)	4.8	1.91	CGU(R)	1.2	1.23
CUC(L)	0	0	CCC(P)	0.7	0.31	CAC(H)	0.2	0.09	CGC(R)	0	0
CUA(L)	1.2	0.17	CCA(P)	2.5	1.1	CAA(Q)	4.5	1.93	CGA(R)	2.6	2.62
CUG(L)	0	0	CCG(P)	0	0	CAG(Q)	0.2	0.07	CGG(R)	0.2	0.15
AUU(I)	34	1.9	ACU(T)	6	2.11	AAU(N)	18.2	1.87	AGU(S)	1.7	0.53
AUC(I)	1.8	0.1	ACC(T)	0.7	0.24	AAC(N)	1.2	0.13	AGC(S)	0	0
AUA(M)	20.5	1.84	ACA(T)	4.6	1.62	AAA(K)	7.9	1.87	AGA(S)	6.6	2.08
AUG(M)	1.8	0.16	ACG(T)	0.1	0.03	AAG(K)	0.5	0.13	AGG(S)	0.5	0.15
GUU(V)	5.1	2.02	GCU(A)	5.3	2.42	GAU(D)	4.2	1.75	GGU(G)	3.5	0.95
GUC(V)	0.2	0.09	GCC(A)	0.6	0.28	GAC(D)	0.6	0.25	GGC(G)	0.2	0.06
GUA(V)	4.7	1.86	GCA(A)	2.8	1.3	GAA(E)	5.4	1.89	GGA(G)	8.9	2.39
GUG(V)	0.1	0.03	GCG(A)	0	0	GAG(E)	0.3	0.11	GGG(G)	2.2	0.6
Average# codons=287											
Codon usage of protein genes in *Timelaea maculata* mitogenome											
Domain: Data											
Codon	Count	RSCU	Codon	Count	RSCU	Codon	Count	RSCU	Codon	Count	RSCU
UUU(F)	28.1	1.89	UCU(S)	9	2.84	UAU(Y)	13.2	1.84	UGU(C)	2.7	1.89
UUC(F)	1.6	0.11	UCC(S)	0.7	0.22	UAC(Y)	1.2	0.16	UGC(C)	0.2	0.11
UUA(L)	35.7	5.08	UCA(S)	7.2	2.26	UAA(*)	0	0	UGA(W)	7.2	1.96
UUG(L)	1.2	0.18	UCG(S)	0.1	0.02	UAG(*)	0	0	UGG(W)	0.2	0.04
CUU(L)	2.5	0.36	CCU(P)	5.2	2.34	CAU(H)	4.8	1.88	CGU(R)	1.3	1.28
CUC(L)	0.3	0.04	CCC(P)	0.9	0.41	CAC(H)	0.3	0.12	CGC(R)	0.2	0.15
CUA(L)	2.3	0.33	CCA(P)	2.7	1.21	CAA(Q)	4.4	1.9	CGA(R)	2.2	2.19
CUG(L)	0.1	0.01	CCG(P)	0.1	0.03	CAG(Q)	0.2	0.1	CGG(R)	0.4	0.38
AUU(I)	33.7	1.87	ACU(T)	5.9	2.17	AAU(N)	18	1.81	AGU(S)	2.2	0.71
AUC(I)	2.4	0.13	ACC(T)	0.9	0.34	AAC(N)	1.8	0.19	AGC(S)	0.1	0.02

AUA(M)	20.1	1.83	ACA(T)	4.1	1.49	AAA(K)	7.4	1.81	AGA(S)	6	1.9
AUG(M)	1.9	0.17	ACG(T)	0	0	AAG(K)	0.8	0.19	AGG(S)	0.1	0.02
GUU(V)	5.2	2.13	GCU(A)	5.5	2.41	GAU(D)	4.5	1.84	GGU(G)	4.1	1.1
GUC(V)	0.1	0.03	GCC(A)	0.8	0.34	GAC(D)	0.4	0.16	GGC(G)	0.3	0.08
GUA(V)	4.3	1.75	GCA(A)	2.8	1.25	GAA(E)	5.5	1.95	GGA(G)	8.6	2.32
GUG(V)	0.2	0.09	GCG(A)	0	0	GAG(E)	0.2	0.05	GGG(G)	1.8	0.5
Average# codons=286											
Codon usage of protein genes in *Chitoria ulupi* mitogenome											
Domain: Data											
Codon	Count	RSCU	Codon	Count	RSCU	Codon	Count	RSCU	Codon	Count	RSCU
UUU(F)	25.9	1.8	UCU(S)	8.2	2.59	UAU(Y)	13	1.83	UGU(C)	2.3	1.71
UUC(F)	2.8	0.2	UCC(S)	0.8	0.27	UAC(Y)	1.2	0.17	UGC(C)	0.4	0.29
UUA(L)	33.5	4.82	UCA(S)	6.6	2.08	UAA(*)	0	0	UGA(W)	6.8	1.89
UUG(L)	1.5	0.22	UCG(S)	0.7	0.22	UAG(*)	0	0	UGG(W)	0.4	0.11
CUU(L)	3.4	0.49	CCU(P)	5.1	2.24	CAU(H)	4.3	1.67	CGU(R)	1.2	1.11
CUC(L)	0.5	0.07	CCC(P)	1.4	0.61	CAC(H)	0.8	0.33	CGC(R)	0.2	0.15
CUA(L)	2.8	0.41	CCA(P)	2.6	1.15	CAA(Q)	4.5	1.93	CGA(R)	2.6	2.52
CUG(L)	0	0	CCG(P)	0	0	CAG(Q)	0.2	0.07	CGG(R)	0.2	0.22
AUU(I)	32.7	1.91	ACU(T)	6.8	2.35	AAU(N)	17	1.74	AGU(S)	2.2	0.68
AUC(I)	1.5	0.09	ACC(T)	0.8	0.27	AAC(N)	2.5	0.26	AGC(S)	0.2	0.05
AUA(M)	20	1.76	ACA(T)	3.8	1.33	AAA(K)	6.8	1.78	AGA(S)	6.7	2.11
AUG(M)	2.7	0.24	ACG(T)	0.2	0.05	AAG(K)	0.8	0.22	AGG(S)	0	0
GUU(V)	6.5	2.15	GCU(A)	5.4	2.28	GAU(D)	4.5	1.73	GGU(G)	4.5	1.18
GUC(V)	0.4	0.13	GCC(A)	0.8	0.36	GAC(D)	0.7	0.27	GGC(G)	0.2	0.06
GUA(V)	4.8	1.62	GCA(A)	3.2	1.33	GAA(E)	4.7	1.69	GGA(G)	7.8	2.06
GUG(V)	0.3	0.1	GCG(A)	0.1	0.03	GAG(E)	0.8	0.31	GGG(G)	2.6	0.69
Average# codons=286											
Codon usage of protein genes in *Euripus nyctelius* mitogenome											
Domain: Data											
Codon	Count	RSCU	Codon	Count	RSCU	Codon	Count	RSCU	Codon	Count	RSCU
UUU(F)	26.4	1.77	UCU(S)	8.6	2.77	UAU(Y)	12.3	1.76	UGU(C)	2.7	1.75
UUC(F)	3.4	0.23	UCC(S)	1.4	0.44	UAC(Y)	1.7	0.24	UGC(C)	0.4	0.25
UUA(L)	31	4.59	UCA(S)	6.2	1.98	UAA(*)	0	0	UGA(W)	6.8	1.87
UUG(L)	2.6	0.39	UCG(S)	0.2	0.07	UAG(*)	0	0	UGG(W)	0.5	0.13
CUU(L)	3.5	0.51	CCU(P)	3.8	1.69	CAU(H)	3.9	1.55	CGU(R)	1.1	1.1
CUC(L)	0.3	0.05	CCC(P)	1.8	0.79	CAC(H)	1.2	0.45	CGC(R)	0.1	0.08
CUA(L)	2.9	0.43	CCA(P)	3.2	1.41	CAA(Q)	4.5	1.9	CGA(R)	2.8	2.82
CUG(L)	0.2	0.03	CCG(P)	0.2	0.1	CAG(Q)	0.2	0.1	CGG(R)	0	0
AUU(I)	34.8	1.86	ACU(T)	6	2.15	AAU(N)	18	1.81	AGU(S)	1.8	0.59
AUC(I)	2.7	0.14	ACC(T)	0.8	0.28	AAC(N)	1.8	0.19	AGC(S)	0	0
AUA(M)	18.8	1.84	ACA(T)	4.2	1.52	AAA(K)	7.2	1.72	AGA(S)	6.6	2.12
AUG(M)	1.7	0.16	ACG(T)	0.2	0.06	AAG(K)	1.2	0.28	AGG(S)	0.1	0.02
GUU(V)	5.5	2.15	GCU(A)	5.3	2.4	GAU(D)	4.5	1.74	GGU(G)	3.8	1.01
GUC(V)	0.3	0.12	GCC(A)	0.4	0.17	GAC(D)	0.7	0.26	GGC(G)	0.4	0.1
GUA(V)	4	1.55	GCA(A)	2.9	1.32	GAA(E)	4.4	1.63	GGA(G)	8.5	2.21
GUG(V)	0.5	0.18	GCG(A)	0.2	0.1	GAG(E)	1	0.37	GGG(G)	2.6	0.68
Average# codons=285											
Codon usage of protein genes in *Herona marathus* mitogenome											
Domain: Data											
Codon	Count	RSCU	Codon	Count	RSCU	Codon	Count	RSCU	Codon	Count	RSCU
UUU(F)	27.2	1.85	UCU(S)	8.3	2.69	UAU(Y)	13.2	1.81	UGU(C)	2.4	1.68

UUC(F)	2.2	0.15	UCC(S)	1.1	0.35	UAC(Y)	1.4	0.19	UGC(C)	0.5	0.32
UUA(L)	33	4.76	UCA(S)	6.5	2.09	UAA(*)	0	0	UGA(W)	6.5	1.79
UUG(L)	3.3	0.48	UCG(S)	0.3	0.1	UAG(*)	0	0	UGG(W)	0.8	0.21
CUU(L)	2.4	0.34	CCU(P)	4.5	1.92	CAU(H)	4.3	1.65	CGU(R)	1.3	1.28
CUC(L)	0.2	0.03	CCC(P)	1.8	0.76	CAC(H)	0.9	0.35	CGC(R)	0	0
CUA(L)	2.6	0.38	CCA(P)	2.9	1.26	CAA(Q)	4.5	1.9	CGA(R)	2.6	2.57
CUG(L)	0.1	0.01	CCG(P)	0.2	0.07	CAG(Q)	0.2	0.1	CGG(R)	0.2	0.15
AUU(I)	34.8	1.86	ACU(T)	5.7	1.97	AAU(N)	17.1	1.72	AGU(S)	1.8	0.6
AUC(I)	2.7	0.14	ACC(T)	1.4	0.48	AAC(N)	2.8	0.28	AGC(S)	0.2	0.05
AUA(M)	18.1	1.73	ACA(T)	4.1	1.41	AAA(K)	7.2	1.84	AGA(S)	6.5	2.09
AUG(M)	2.8	0.27	ACG(T)	0.4	0.13	AAG(K)	0.6	0.16	AGG(S)	0.1	0.02
GUU(V)	4.4	1.81	GCU(A)	4.6	2.02	GAU(D)	4.5	1.76	GGU(G)	5.1	1.34
GUC(V)	0.2	0.06	GCC(A)	0.8	0.34	GAC(D)	0.6	0.24	GGC(G)	0.3	0.08
GUA(V)	4.5	1.84	GCA(A)	3.6	1.58	GAA(E)	4.8	1.73	GGA(G)	7.2	1.91
GUG(V)	0.7	0.29	GCG(A)	0.2	0.07	GAG(E)	0.8	0.27	GGG(G)	2.5	0.67
Average# codons=286											
Codon usage of protein genes in *Apatura laverna* mitogenome											
Domain: Data											
Codon	Count	RSCU	Codon	Count	RSCU	Codon	Count	RSCU	Codon	Count	RSCU
UUU(F)	26.6	1.81	UCU(S)	7.5	2.3	UAU(Y)	13	1.82	UGU(C)	2.2	1.61
UUC(F)	2.8	0.19	UCC(S)	1.2	0.35	UAC(Y)	1.3	0.18	UGC(C)	0.5	0.39
UUA(L)	32.8	4.68	UCA(S)	8.2	2.51	UAA(*)	0	0	UGA(W)	6.8	1.89
UUG(L)	3.5	0.49	UCG(S)	0.2	0.05	UAG(*)	0	0	UGG(W)	0.4	0.11
CUU(L)	3.2	0.45	CCU(P)	4.9	2.23	CAU(H)	4	1.55	CGU(R)	1.4	1.36
CUC(L)	0.4	0.05	CCC(P)	0.9	0.42	CAC(H)	1.2	0.45	CGC(R)	0.1	0.08
CUA(L)	2.1	0.3	CCA(P)	2.8	1.29	CAA(Q)	4.4	1.93	CGA(R)	2.5	2.49
CUG(L)	0.2	0.02	CCG(P)	0.2	0.07	CAG(Q)	0.2	0.07	CGG(R)	0.1	0.08
AUU(I)	32.5	1.83	ACU(T)	5.7	2	AAU(N)	17.8	1.83	AGU(S)	2.5	0.75
AUC(I)	2.9	0.17	ACC(T)	1.2	0.43	AAC(N)	1.6	0.17	AGC(S)	0.1	0.02
AUA(M)	19.3	1.77	ACA(T)	4.5	1.57	AAA(K)	6.9	1.73	AGA(S)	6.5	1.99
AUG(M)	2.5	0.23	ACG(T)	0	0	AAG(K)	1.1	0.27	AGG(S)	0.1	0.02
GUU(V)	5.8	2.11	GCU(A)	5.4	2.46	GAU(D)	4.7	1.85	GGU(G)	5.1	1.35
GUC(V)	0.4	0.14	GCC(A)	0.3	0.14	GAC(D)	0.4	0.15	GGC(G)	0.5	0.14
GUA(V)	4.8	1.72	GCA(A)	2.8	1.3	GAA(E)	5.1	1.78	GGA(G)	6.9	1.84
GUG(V)	0.1	0.03	GCG(A)	0.2	0.11	GAG(E)	0.6	0.22	GGG(G)	2.5	0.67
Average# codons=286											
Codon usage of protein genes in *Hestina assimilis* mitogenome											
Domain: Data											
Codon	Count	RSCU	Codon	Count	RSCU	Codon	Count	RSCU	Codon	Count	RSCU
UUU(F)	27.7	1.84	UCU(S)	8.8	2.77	UAU(Y)	13.2	1.86	UGU(C)	2.7	2
UUC(F)	2.5	0.16	UCC(S)	0.9	0.29	UAC(Y)	1	0.14	UGC(C)	0	0
UUA(L)	35.2	5.06	UCA(S)	6.3	1.99	UAA(*)	0	0	UGA(W)	6.8	1.89
UUG(L)	1.4	0.2	UCG(S)	0.2	0.05	UAG(*)	0	0	UGG(W)	0.4	0.11
CUU(L)	3	0.43	CCU(P)	5.5	2.5	CAU(H)	4.5	1.76	CGU(R)	1.1	1.08
CUC(L)	0.1	0.01	CCC(P)	0.5	0.21	CAC(H)	0.6	0.24	CGC(R)	0.2	0.15
CUA(L)	2	0.29	CCA(P)	2.7	1.22	CAA(Q)	4.5	1.9	CGA(R)	2.6	2.62
CUG(L)	0.1	0.01	CCG(P)	0.2	0.07	CAG(Q)	0.2	0.1	CGG(R)	0.2	0.15
AUU(I)	34.8	1.9	ACU(T)	5.8	2.08	AAU(N)	18.5	1.9	AGU(S)	2.1	0.66
AUC(I)	1.8	0.1	ACC(T)	0.8	0.27	AAC(N)	1	0.1	AGC(S)	0.2	0.07
AUA(M)	20.2	1.85	ACA(T)	4.5	1.62	AAA(K)	7.8	1.87	AGA(S)	6.8	2.16
AUG(M)	1.7	0.15	ACG(T)	0.1	0.03	AAG(K)	0.5	0.13	AGG(S)	0	0

GUU(V)	5.4	2.14	GCU(A)	5.3	2.4	GAU(D)	4.5	1.81	GGU(G)	3.4	0.92
GUC(V)	0.2	0.09	GCC(A)	0.7	0.31	GAC(D)	0.5	0.19	GGC(G)	0.2	0.06
GUA(V)	4.2	1.65	GCA(A)	2.8	1.25	GAA(E)	5.2	1.86	GGA(G)	9.5	2.58
GUG(V)	0.3	0.12	GCG(A)	0.1	0.03	GAG(E)	0.4	0.14	GGG(G)	1.6	0.44
Average# codons=286											
Codon usage of protein genes in *Hestina persimilis* mitogenome											
Domain: Data											
Codon	Count	RSCU	Codon	Count	RSCU	Codon	Count	RSCU	Codon	Count	RSCU
UUU(F)	26.6	1.78	UCU(S)	9.2	2.87	UAU(Y)	13	1.86	UGU(C)	2.5	1.88
UUC(F)	3.2	0.22	UCC(S)	1	0.31	UAC(Y)	1	0.14	UGC(C)	0.2	0.12
UUA(L)	34.1	4.99	UCA(S)	6	1.86	UAA(*)	0.1	2	UGA(W)	7.1	1.96
UUG(L)	1.3	0.19	UCG(S)	0	0	UAG(*)	0	0	UGG(W)	0.2	0.04
CUU(L)	3.5	0.52	CCU(P)	5.2	2.32	CAU(H)	4.5	1.76	CGU(R)	1.1	1.08
CUC(L)	0	0	CCC(P)	0.9	0.41	CAC(H)	0.6	0.24	CGC(R)	0.2	0.15
CUA(L)	2	0.29	CCA(P)	2.8	1.26	CAA(Q)	4.3	1.81	CGA(R)	2.7	2.69
CUG(L)	0.1	0.01	CCG(P)	0	0	CAG(Q)	0.5	0.19	CGG(R)	0.1	0.08
AUU(I)	35.6	1.91	ACU(T)	6.1	2.18	AAU(N)	18.5	1.88	AGU(S)	2.5	0.76
AUC(I)	1.7	0.09	ACC(T)	0.6	0.22	AAC(N)	1.2	0.12	AGC(S)	0.2	0.05
AUA(M)	19.6	1.83	ACA(T)	4.5	1.6	AAA(K)	7.6	1.83	AGA(S)	6.9	2.15
AUG(M)	1.8	0.17	ACG(T)	0	0	AAG(K)	0.7	0.17	AGG(S)	0	0
GUU(V)	5.1	2.18	GCU(A)	5.5	2.48	GAU(D)	4.4	1.9	GGU(G)	3.7	0.99
GUC(V)	0.2	0.07	GCC(A)	0.5	0.21	GAC(D)	0.2	0.1	GGC(G)	0.1	0.02
GUA(V)	3.8	1.62	GCA(A)	2.8	1.24	GAA(E)	5.1	1.78	GGA(G)	9.1	2.43
GUG(V)	0.3	0.13	GCG(A)	0.2	0.07	GAG(E)	0.6	0.22	GGG(G)	2.1	0.56
Average# codons=285											
Codon usage of protein genes in *Hestina nama* mitogenome											
Domain: Data											
Codon	Count	RSCU	Codon	Count	RSCU	Codon	Count	RSCU	Codon	Count	RSCU
UUU(F)	25.9	1.83	UCU(S)	9.2	2.87	UAU(Y)	13.5	1.8	UGU(C)	2.2	1.71
UUC(F)	2.4	0.17	UCC(S)	1.2	0.36	UAC(Y)	1.5	0.2	UGC(C)	0.4	0.29
UUA(L)	33.6	4.81	UCA(S)	5.8	1.82	UAA(*)	0.1	2	UGA(W)	6.8	1.87
UUG(L)	2.5	0.36	UCG(S)	0.2	0.07	UAG(*)	0	0	UGG(W)	0.5	0.13
CUU(L)	3	0.43	CCU(P)	4.1	1.78	CAU(H)	4.2	1.64	CGU(R)	0.9	0.91
CUC(L)	0.4	0.06	CCC(P)	1.6	0.71	CAC(H)	0.9	0.36	CGC(R)	0.2	0.15
CUA(L)	2.3	0.33	CCA(P)	3.4	1.48	CAA(Q)	4	1.76	CGA(R)	2.5	2.49
CUG(L)	0.1	0.01	CCG(P)	0.1	0.03	CAG(Q)	0.5	0.24	CGG(R)	0.5	0.45
AUU(I)	31.2	1.8	ACU(T)	5.8	1.91	AAU(N)	15.9	1.65	AGU(S)	2.4	0.74
AUC(I)	3.5	0.2	ACC(T)	1.2	0.4	AAC(N)	3.4	0.35	AGC(S)	0.2	0.07
AUA(M)	18.2	1.68	ACA(T)	5.1	1.66	AAA(K)	6.9	1.82	AGA(S)	6.5	2.04
AUG(M)	3.5	0.32	ACG(T)	0.1	0.03	AAG(K)	0.7	0.18	AGG(S)	0.1	0.02
GUU(V)	7.1	2.47	GCU(A)	4.8	2.19	GAU(D)	4.6	1.74	GGU(G)	5.1	1.35
GUC(V)	0.5	0.19	GCC(A)	0.9	0.42	GAC(D)	0.7	0.26	GGC(G)	0.2	0.06
GUA(V)	3.5	1.21	GCA(A)	3	1.36	GAA(E)	4.3	1.56	GGA(G)	6.3	1.68
GUG(V)	0.4	0.13	GCG(A)	0.1	0.03	GAG(E)	1.2	0.44	GGG(G)	3.4	0.9
Average# codons=285											
Relative synonymous codon usage is given in parantheses following the codon frequency.											

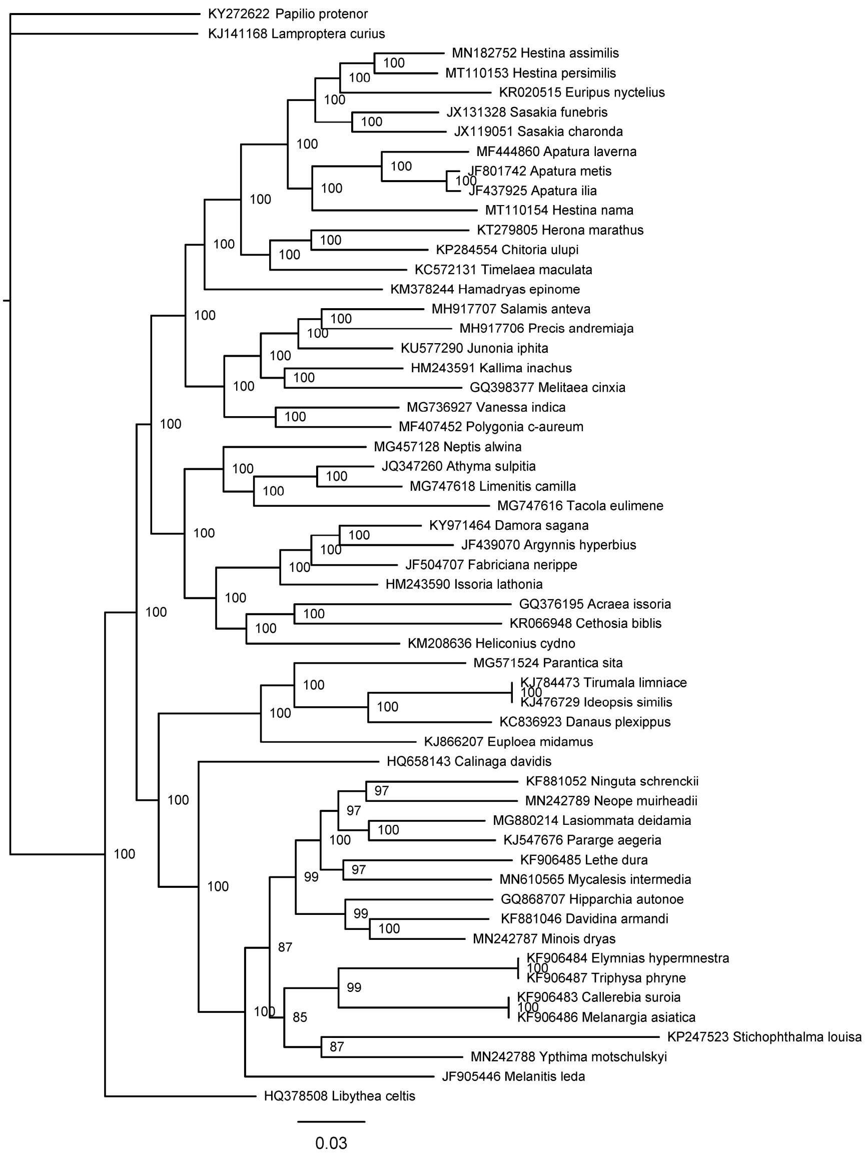

Figure S1. Phylogenetic relationships by BI analysis based on the 13PCGs.

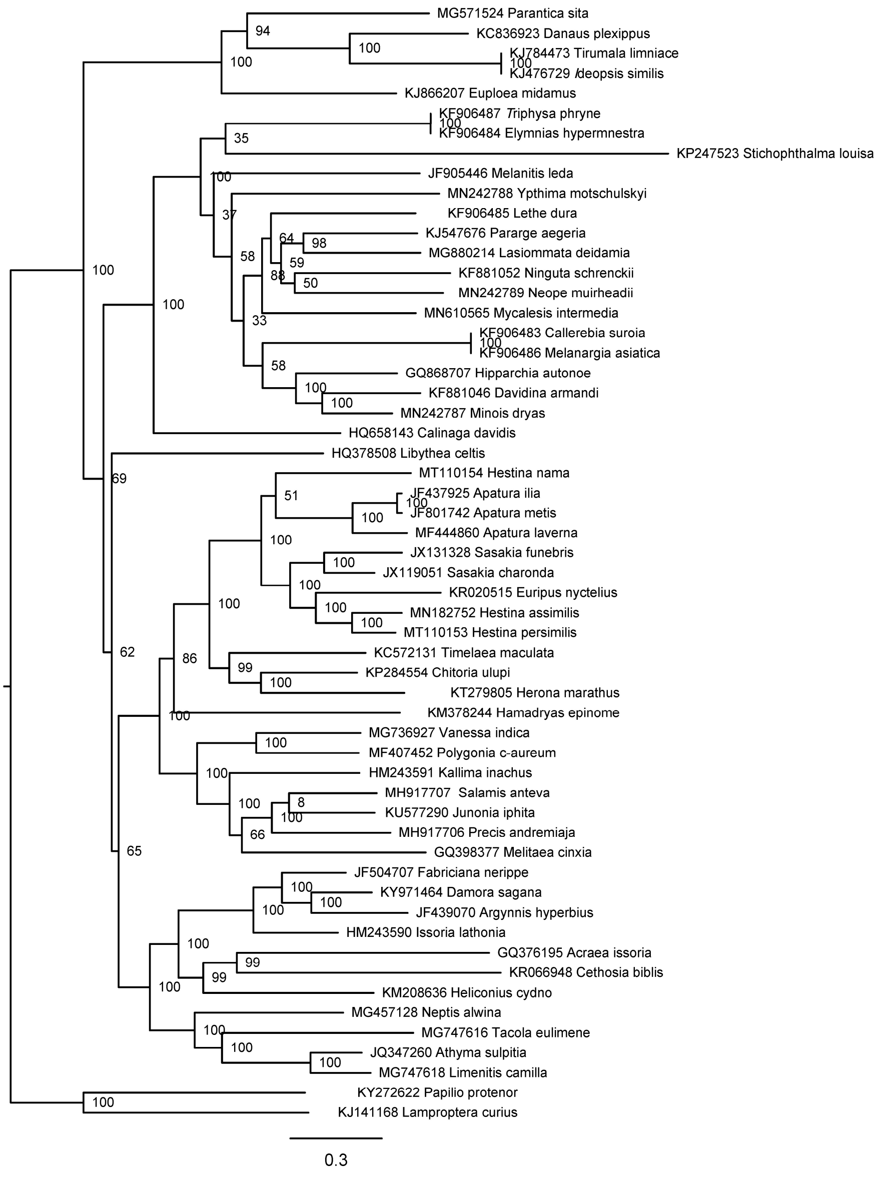

Figure S2. phylogenetic relationships by ML analysis based on the 13PCGs.
